# Choosing the right density for a concentrated protein system like gluten in a coarse-grained model

**DOI:** 10.1007/s00249-023-01667-8

**Published:** 2023-06-28

**Authors:** Łukasz Mioduszewski

**Affiliations:** Faculty of Mathematics and Natural Sciences, Cardinal Stefan Wyszyński University, Wóycickiego 1/3, 01-938 Warsaw, Poland

## Abstract

Large coarse-grained simulations are often conducted with an implicit solvent, which makes it hard to assess the water content of the sample and the effective concentration of the system. Here the number and the size of cavities and entanglements in the system, together with density profiles, are used to asses the homogeneity and interconnectedness of gluten. This is a continuation of an earlier article, "Viscoelastic properties of wheat gluten in a molecular dynamics study" (Mioduszewski and Cieplak 2021b). It turns out there is a wide range of densities (between 1 residue per cubic nanometer and 3 residues/nm$^3$) where the system is interconnected, but not homogeneous: there are still large empty spaces, surrounded by an entangled protein network. Those findings should be of importance to any coarse-grained simulation of large protein systems.

**Supplementary Information:**

The online version contains supplementary material available at 10.1007/s00249-023-01667-8.

## Introduction

Coarse-grained simulations are very helpful in reaching timescales and system sizes unreachable by other methods (Underhill and Doyle 2004). Coarse-grained protein models have a very wide range of applications (Kmiecik et al. 2016; Rief 1997), especially in the emerging field of studying the phase separation in dense protein systems (Dignon et al. 2018; Mioduszewski and Cieplak 2020). The liquid–liquid phase separation is the process responsible for formation of many cellular membraneless organelles, like stress granules, Cajal bodies (Shapiro et al. 2021), and nucleoli (Caragine et al. 2018). Proteins that undergo phase separation are often intrinsically disordered or have intrinsically disordered regions (Shapiro et al. 2021; Posey et al. 2018). Some of them are involved in various diseases, like the Fused in Sarcoma (FUS) protein (Shapiro et al. 2021; Ahlers et al. 2021) or the proteins containing polyglutamine-rich regions 0. Sometimes the phase separation happens between a liquid and a solid phase (Mioduszewski and Cieplak 2020; Peskett et al. 2018; Patel et al. 2015). Such a process is also important in the creation of virus capsids (Iserman et al. 2020; Marek et al. 2022). Knowing exactly the protein concentration in simulations of dense protein systems may be very important, the phase separation can happen only for a specific range of protein concentrations (Shapiro et al. 2021). It is also important to distinguish between the dense protein liquid and the solid phases. The solvent inside the dense protein phase exhibits different properties from the solvent in the dilute phase (Ahlers et al. 2021). Finally, gluten protein solutions have very different viscoelastic properties depending on the water content 0. However, the fastest and most computationally feasible coarse-grained models use an implicit solvent (Kmiecik et al. 2016; Dignon et al. 2018; Mioduszewski and Cieplak 2020), which makes it hard to calculate the concentration of the system: water molecules are missing and only the protein density is accessible. Thus, a question arises: what is the effective protein concentration? Using an explicit solvent slows down the simulations, so only small fragments of the protein condensate may be studied (Dignon et al. 2019; Paloni et al. 2020). To study the system on a scale where protein concentration plays a role, coarse-grained implicit solvent models are usually chosen (Mioduszewski and Cieplak 2020; Dignon et al. 2019; Regy et al. 2021; Tesei et al. 2021). All atom simulations with explicit solvent are also possible (Rauscher and Pomès 2017), but they are limited to very short time scales (Dignon et al. 2019; Rauscher and Pomès 2017).

Here I would like to present some quantities that correlate with the protein density $\rho$ (the number of amino acid residues per cubic nanometer, measured in nm$^{-3}$), as exemplified by the simulations of gluten proteins, which are intrinsically disordered (Mioduszewski and Cieplak 2021b). The main findings of our gluten simulations and their detailed methodology are published in another article (Mioduszewski and Cieplak 2021b), but the problem of choosing the correct density $\rho$ is only mentioned there (Mioduszewski and Cieplak 2021a). The protein density $\rho$ can be easily calculated by dividing the number of residues by the volume of the simulation box, but it is not the true density of the system: it does not take into account the solvent molecules. The protein concentration *c* is strongly correlated with $\rho$, but they do not have to be directly proportional: for sufficiently high $\rho$, the system ceases to be a protein solution and starts to be a gel or even a solid, where single water molecules are trapped in cavities between the proteins. A good variable for describing this transition is the volume of the biggest cavity $V^C_{max}$ computed near the end of the simulation (cavities in a protein system can be determined for any.pdb file by the Spaceball algorithm Chwastyk et al. 2014). For low densities, $V^C_{max}$ rises with $\rho$ because a cavity is not the same as free space. It needs to be surrounded by proteins, and in low $\rho$, the protein network is too sparse to form a cavity. Then, after reaching a maximum, $V^C_{max}$ decreases for large $\rho$ values due to the increased compactness of the system (see Fig. 3). This is an example of a value that changes non-monotonically with $\rho$ and helps to find a point where the system changes from a solution to a possible gel: the maximum in the $V^C_{max}(\rho )$ plot. The cavities that are empty in the coarse-grained model would be filled with the solvent in reality, so if their volume decreases, it means the effective concentration of the system gets higher. However, if the $V^C_{max}(\rho )$ curve is flat near its maximum, the threshold $\rho$ value has to be determined by different methods.

In this article, we show other, previously not discussed, but equally helpful measures to determine the effective protein concentration. These measures will be shown in the Results section of this article. In every section, density $\rho$ always means the number of residues per cubic nanometer and not the real density of the system. The Results are preceded by the Methods section that recapitulates the simulation protocol from earlier works (Mioduszewski and Cieplak 2020, 2021) in a condensed manner. Finally, the Conclusions section summarizes the different approaches presented here and draws the connection between the protein density $\rho$ (the variable we can measure during simulations) and the effective protein concentration *c* (the variable we want to establish).

## Methods

### Gluten composition

Gluten proteins can be divided into gliadins (soluble in a 50 % alcohol solution) and glutenins (insoluble in a 50 % alcohol solution due to formation of inter-chain disulfide bonds and entanglements between longer chains) (Lafiandra et al. 2004; Wieser 2007). Each of these protein groups comprises many different sequences, over 100 in total (Wieser 2007). The simulated gluten system is made from 4271 residues (Mioduszewski and Cieplak 2021a), so we chose the most representative sequences of gliadins and glutenins in the right proportions (exact composition can be found in the S1 text of our other article, Mioduszewski and Cieplak 2021a). The amino acid most common in gluten is glutamine (over 30 %), followed by proline and glycine (Wieser 2007).

### The DSB model

Our coarse-grained Dynamic Structure-Based (DSB) model (Mioduszewski and Cieplak 2018) uses a one-bead-per-residue coarse-grained representation with Langevin dynamics (see Equation 1) that enables milisecond-scale simulations of large protein systems, while preserving their amino acid specificity. The mass of each residue is set to the average amino acid mass *m*.

1 $$\begin{aligned} m\frac{d^2\vec {r}}{d^2t}=\vec {F}-\gamma \frac{dr}{dt}+\vec {\Gamma } \end{aligned}$$

In the equation, *r* is the position of a residue, *t* is time, *F* is the force obtained from the force field, $\gamma =2m/\tau$ (time unit $\tau \approx 1$ ns is equal to 200 simulation steps), and the thermal white noise $\Gamma$ has variance $\sigma ^2=2\gamma k_B T$. Thermostat is implemented only via this white noise term in the Langevin equation. The $\gamma$ value corresponds to overdamped dynamics (Szymczak and Cieplak 2006).

We use the energy unit $\epsilon$ where $0.3\epsilon \lesssim k_BT \lesssim 0.38\epsilon$ for room temperature (Sułkowska and Cieplak 2008; Mioduszewski et al. 2020). Residues in a chain are connected by a harmonic potential with the spring constant $k=50$ Å$^{-2}\cdot \epsilon$ and a minimum at $r_b=3.8$ Å (Korkut and Hendrickson 2009; Poma et al. 2015). The chain stiffness is kept by the bond angle and dihedral potentials obtained from a random coil library (Ghavani et al. 2013). The excluded volume is ensured by Lennard-Jones (L-J) potential cut at $r_{o}=5$ Å  (so that $V_r(r_{i,j}\ge r_{o})=0$), where $r_{i,j}$ is the distance between residues *i* and *j*.

2 $$\begin{aligned} V_r(r_{i,j}\le r_{o})=\epsilon \left( \left( \frac{r_0}{r_{i,j}}\right) ^{12} - 2 \left( \frac{r_0}{r_{i,j}}\right) ^6 + 1 \right) \end{aligned}$$

For $j=i+2$, interactions are always described by $V_r$.

In order to distinguish backbone–backbone (bb), backbone–side chain (bs), and side chain–side chain (ss) contacts in a one-bead-per-residue model, we compute the normal and binormal vectors based on the coordinate system made by three consecutive beads ($i-1$, *i*, $i+1$): the negative normal vector points approximately in the side chain direction and the binormal points in the direction where a backbone hydrogen bond can be made. An attractive interaction can be turned on only for specific directions of these vectors. Each type of amino acid has a maximum coordination number $n_c$ for its virtual, backbone, and side chain (Mioduszewski and Cieplak 2018). Each pair of amino acids has a different equilibrium distance at the minimum of the Lennard–Jones potential corresponding to an ss contact between them. That distance is 5 Å for bb contacts, and 6.8 Å for bs contacts. Each type of contact is modeled by the classic 6–12 Lennard–Jones (L–J) potential with the depth $\epsilon$ and a minimum $r_0$ dependent on the type of interaction. If the criteria for the geometry, number of contacts, and distance are met, the potential is turned on quasi-adiabatically (with time scale 10 $\tau$). The contact is turned off in the same manner if the distance between the residues exceeds $3/2 \cdot r_0/2^{1/6}$.

We distinguish disulfide bonds using a L–J potential with the depth 4$\epsilon$. These bonds can form and rupture dynamically similarly to the ss contacts (Mioduszewski and Cieplak 2021b), but one cysteine residue can make only one such bond.

Electrostatics are governed by Debye–Hueckel potential with relative permittivity dependent on length (Tozzini et al. 2007): $\kappa =4\,$Å$^{-1}\, r$. Some gluten proteins are thought to be partially structured, so some of the contacts are always attractive. We incorporated available information about possible structured parts of gluten using a structure-based Go model for those parts (Sułkowska and Cieplak 2008). The structures of those parts were generated by the ITASSER server (Yang et al. 2015) and used to create contact maps that determine which contacts are always attractive. The L–J potential describing them has a minimum corresponding to the distance between the C$_{\alpha }$ atoms in the predicted structure. Residues listed in the contact map can still make, disordered contacts with the residues outside the contact map.

The inter-chain interactions work in the same way as the intra-chain: they can also be backbone–backbone, backbone–side chain, or side chain–side chain. Inter-chain disulfide bonds and electrostatic interactions are also the same as their intra-chain counterparts. The only difference occurs for the partially structured regions, which have additional Go model contacts that are only intra-chain.

We tried modifying the DSB model to take into account the solvation effect: solvent molecules compete for contacts with residues, so residues surrounded by other residues should form more contacts, because they are shielded from the solvent. Conversely, more exposed residues should form less contacts. The number of neighbors $n_n$ was computed as the number of all residues closer than 0.75 nm to a given residue. If $n_n$ is higher than a given threshold $n_t$, the maximum coordination number $n_c$ for a given residue changes by 1. However, for the range of $n_t$ from 0 to 10, the results were the same (or the change was statistically insignificant), so we decided to not modify the model (setting $n_t$ to 0).

The experimental validation of the DSB model is described in detail in other works (Mioduszewski and Cieplak 2018; Mioduszewski et al. 2020). It was parameterized and tested based on a set of 23 different intrinsically disordered proteins (Mioduszewski et al. 2020). Here we use the, original version of the model, as described in the first article (Mioduszewski and Cieplak 2018). Additionally, the dynamic Young modulus of gluten corresponds well to the experimental data (see Fig. 5 in our earlier article, Mioduszewski and Cieplak 2021b). The technical details of the implementation are also described elsewhere (Mioduszewski et al. 2023).

### Simulation protocol

We use periodic boundary conditions in two directions (X and Y) and solid walls (with potential $V_{wall}(r_{residue-wall})=\frac{-\epsilon }{9(r/0.5 nm)^9}$) in the third direction (Z) (Cieplak and Robbins 2010). Our simulations cannot start from the native structure, because of the lack of it, so the starting conformations are generated as a self-avoiding random walk, and then evolve according to the molecular dynamics potential, which was shown to correctly recreate properties of a set of intrinsically disordered proteins (Mioduszewski and Cieplak 2018). The initial density $\rho _{start}<0.1$ nm$^{-3}$ is chosen so that each randomly placed chain is not in contact with other chains.

After equilibrating the system for 200,000 $\tau$, the box is squeezed in every direction with a speed 0.02 Å$/\tau$, until the target density $\rho _0$ is accomplished. The box is a square, the side length is chosen to get the desired density (for example for $\rho =3.5$ nm${^-3}$ the side length is $\root 3 \of {4271/3.5}$ nm $\approx 10.7$ nm). The change of the box size happens slow enough for the system to be described as a NVT ensemble. Then the system has another 200,000 $\tau$ for equilibration. After the equilibration, the walls along the Z direction start to attract the proteins with Lenard–Jones potential with the depth $4\epsilon$ (the same as for disulfide bonds), again in a quasi-adiabatic contact-switching manner (Mioduszewski and Cieplak 2021b). The details of the adhesion process are also very important and we devoted an entire article to them (Mioduszewski and Cieplak 2021c). After another (third) equilibration, when the attraction is already turned on, the box shape changes periodically (with the period 40,000 $\tau$) to recreate the shearing and normal strain (the box is no longer a square, but a parallelepiped - more details can be found in Mioduszewski and Cieplak 2021b).

### Calculating the number and size of cavities and entanglements

The number and the size of cavities are computed using the Spaceball algorithm, which fills the space with a grid of balls (hence the name) and then rotates the grid to remove the balls outside the proteins, so at the end, only those in cavities are left (Chwastyk et al. 2016). The ball radius used in this work is 1.9 Å (3.8 Å is a length unit for a coarse-grained system—the commonly used radius of water molecule would be impractical in a coarse-grained representation), and the grid spacing is 1 Å, the number of rotations was 1. We used a special stand-alone version of Spaceball (Chwastyk et al. 2016).

**Fig. 1:**
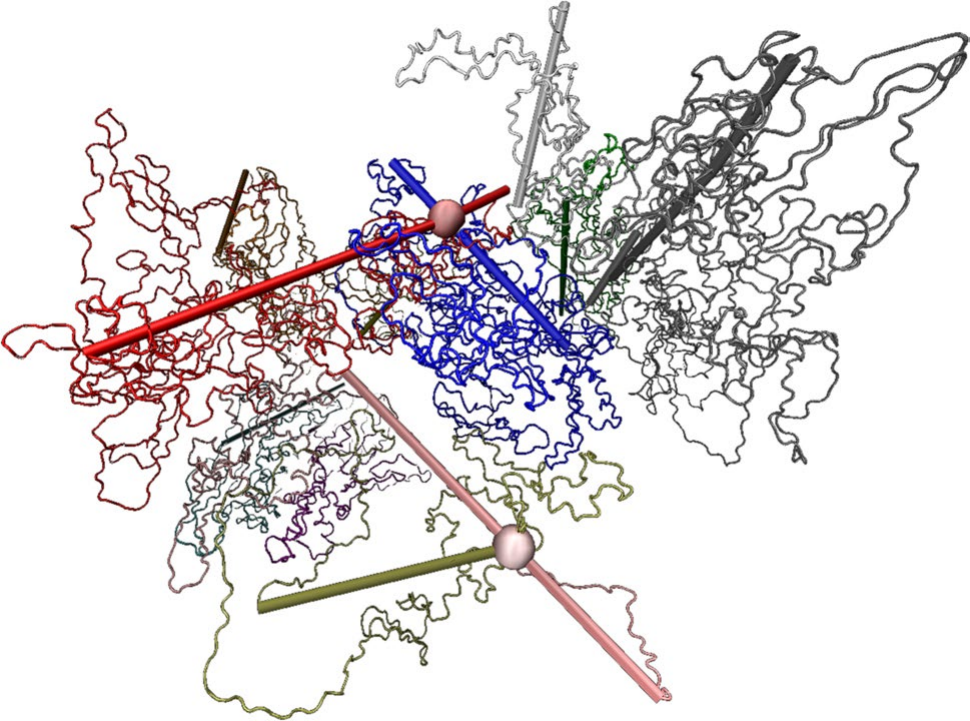
Illustration of the Z1 algorithm results: each protein chain has a rod that connects its termini. If two chains are entangled, their rods are bent and the intersection point is marked by a ball. Each color corresponds to a different chain

The number of (pairwise) entanglements is calculated by the Z1 algorithm, which keeps the protein termini fixed in place and shrinks each protein chain to check if it can form a line connecting its termini without intersecting other chains (then it is not entangled) (Kröger 2005). If these lines intersect and form a kink, two proteins are entangled (see Fig. 1 for an example).

Each simulation was repeated at least 3 times with different random initial conditions to get an average value with error bars. The number of repeats is different for different densities (higher densities require significantly longer computation times).

## Results

### Kinetics

**Fig. 2:**
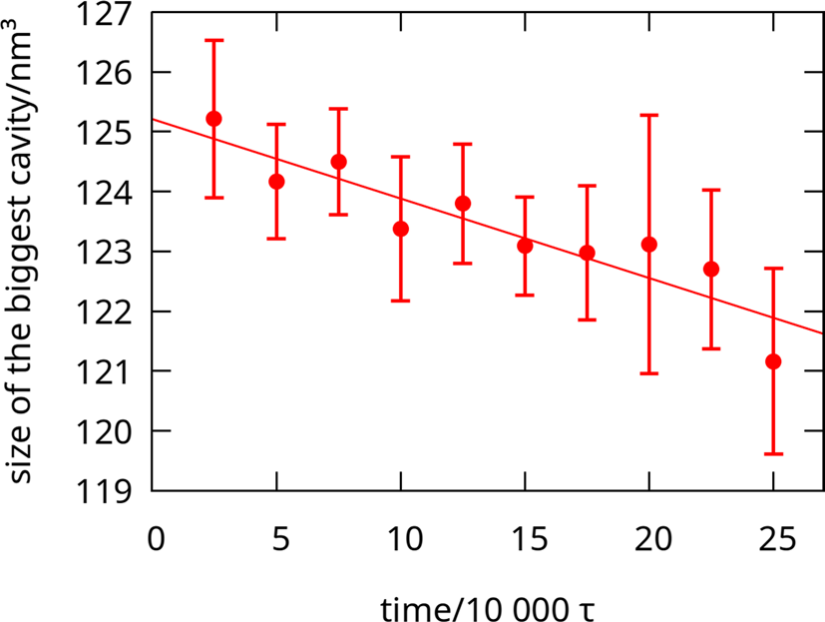
Size of the biggest cavity $V^C_{max}$ during oscillations (averaged over pulling and shearing oscillations) for $\rho =3.5$ nm$^{-3}$. The fitted line is just to guide the eye

The results shown here are computed after periodic deformation of the box, unless stated otherwise. This is because applying strain changes properties of the system and strengthens the protein network (Mioduszewski and Cieplak 2021b). In Fig. 2, we see that during oscillations, the size of the biggest cavity lowers (the system becomes more homogeneous). The number of entanglements also seems to rise (proteins become more interconnected), but the data is too noisy to show it. A comparison of the number of entanglements before and after oscillations (Fig. 2 in our earlier article: Mioduszewski and Cieplak 2021b) shows a clear increase. Because real systems have large relaxation times (up to hours, see Singh and MacRitchie 2001), the mechanical deformation may speed up, solidifying the system, which would be otherwise unreachable in the time scales allowed in coarse-grained simulations.

**Fig. 3:**
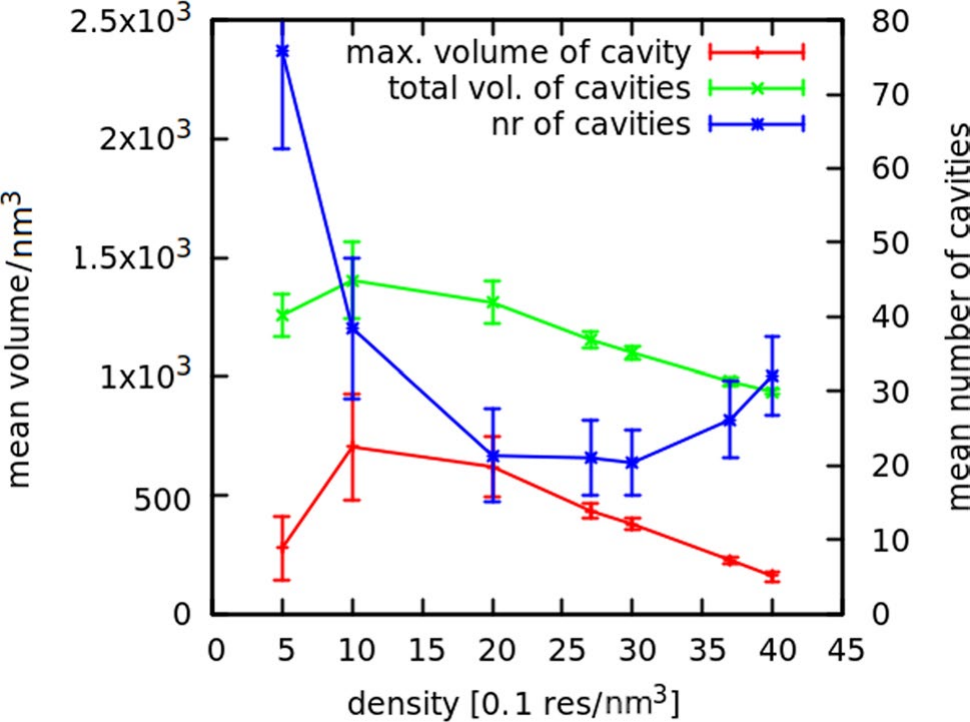
Density dependence of the number and size of cavities calculated with Spaceball (Chwastyk et al. 2016)

### Density profiles and cavities

**Fig. 4:**
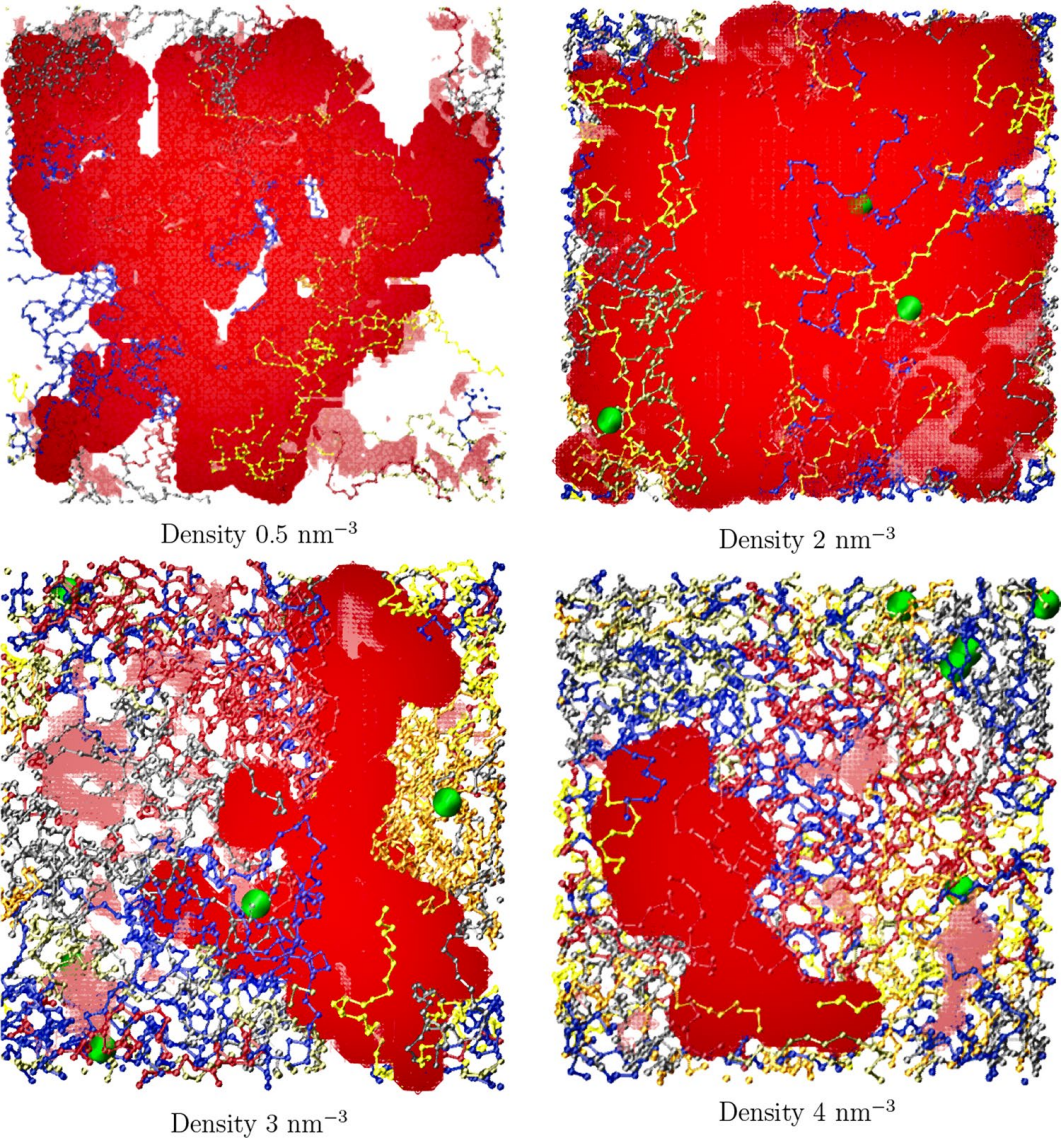
Snapshots of the system for different densities, taken after equilibration. Each chain has a different color, the biggest cavity is shown in red, other cavities in pink. Entanglement points are shown as green spheres

**Fig. 5:**
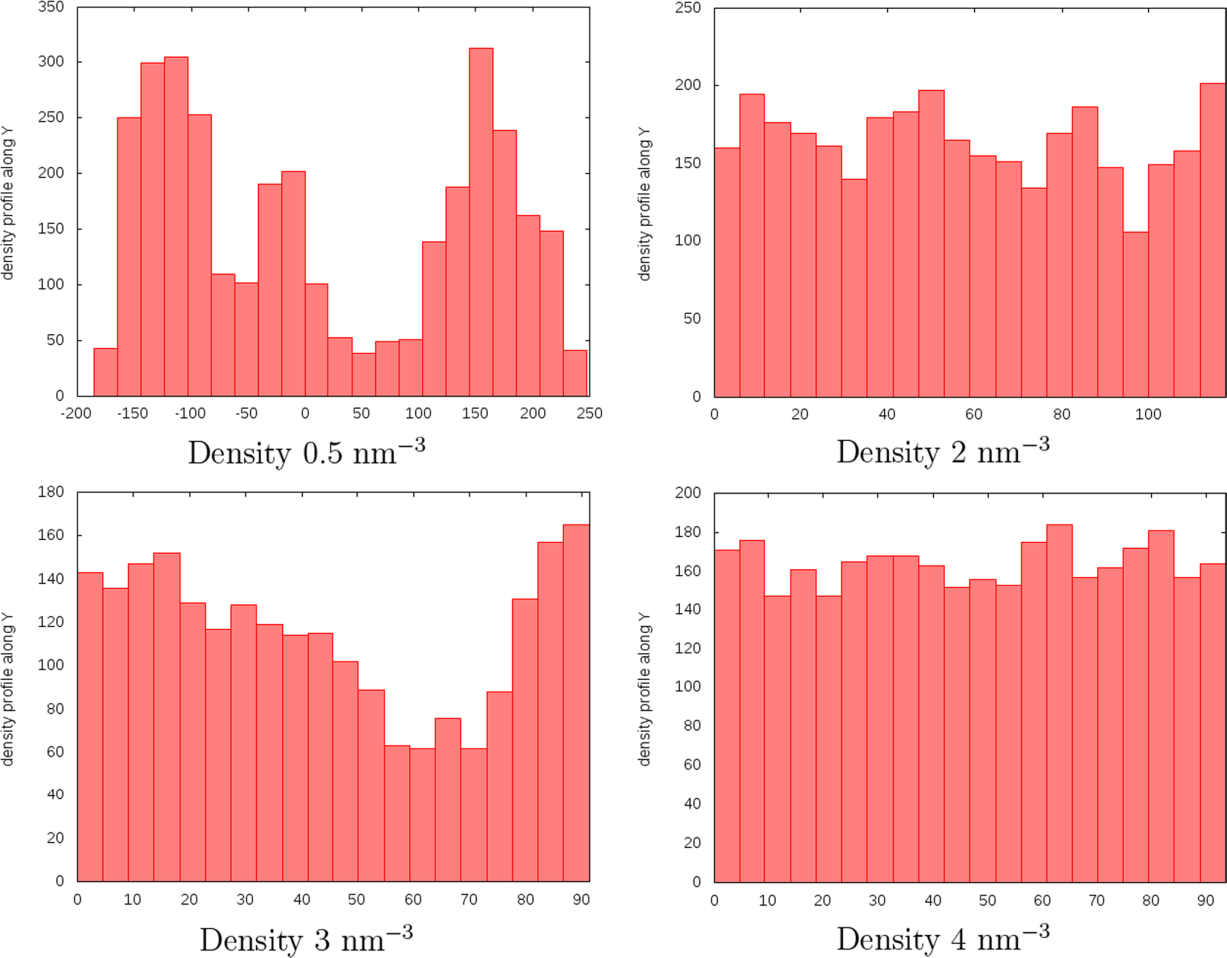
Number of residues along the Y axis (in Å), summed over X and Z

Figure 3 shows that the volume of the biggest cavity and the total volume of cavities rise for $\rho < 1$ nm$^{-3}$. It should be concluded that below the 1 nm$^{-3}$ threshold, the protein chains do not form a single network, and lowering the box size allows more chains to come in contact and form bigger cavities. For larger densities, the system may be regarded as interconnected, because the volumes decrease. The number of the cavities rapidly decreases in low densities, because smaller cavities merge into bigger ones as the proteins come closer to each other. This merging process stops at $\rho \approx 2$ nm$^{-3}$. For $\rho > 3$ nm$^{-3}$, the number of cavities starts to increase again because squeezing the proteins causes the collapse of large cavities, which are divided into many smaller ones. Only at this point, the system can be regarded as homogeneous: this is the first justification of choosing 3.5 nm$^{-3}$ as the correct gluten density. This is further validated by the density profiles on Fig. 5 and illustrated by Fig. 4.

We studied densities ranging from 0.5 nm$^{-3}$ to 4 nm$^{-3}$, to find out that even for the density 3 nm$^{-3}$, the system is still not quite homogeneous (see Fig. 5) and at least the density 3.5 nm$^{-3}$ should be used. This proves that the number of cavities is a better descriptor of the system homogeneity than their volume (see Fig. 3).

### Chain compactness and entanglements

**Fig. 6:**
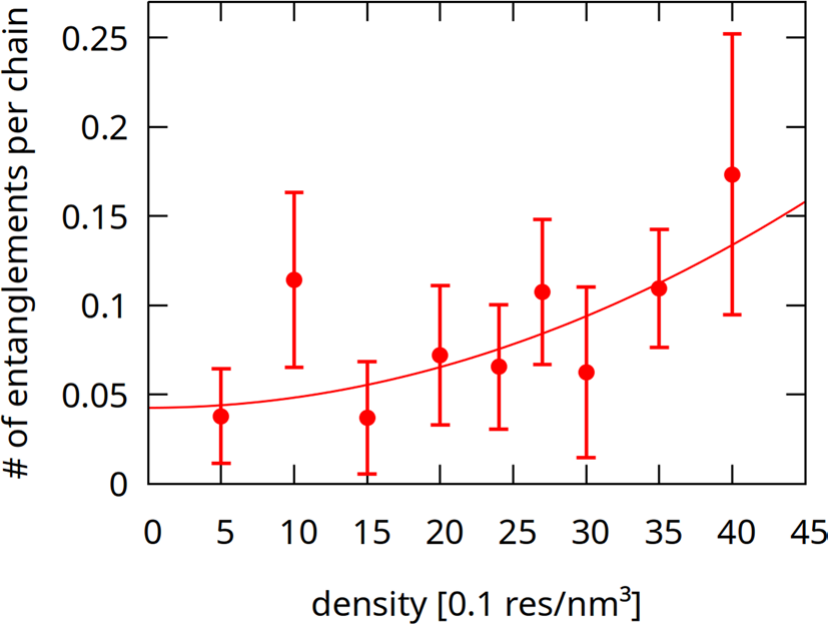
Density dependence of the number of entanglements calculated with Z1 algorithm (Kröger 2005). The fitted quadratic curve is just to guide the eye

**Fig. 7:**
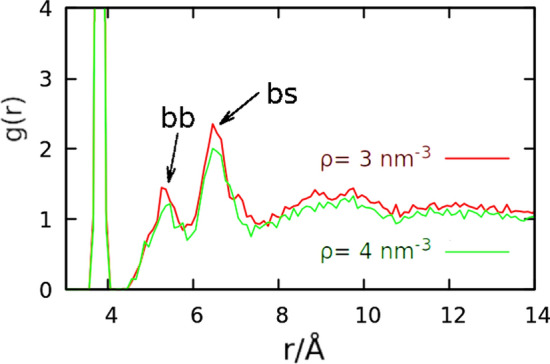
Radial distribution functions for $\rho$ equal to 3 nm$^{-3}$ and 4 nm$^{-3}$. The peaks from backbone–backbone (bb) and backbone–side chain (bs) contacts are marked

The average number of entanglements (see Fig. 6) rises for higher densities: gluten proteins are not globular (Anjum et al. 2007; Matsushima et al. 1990; Shewry et al. 2002) but form an entangled network (Singh and MacRitchie 2001). This network is strengthened after mechanical deformation, which recreates (on a molecular level) the conditions encountered in the dough mixing process. The mixed dough must have high enough concentration of gluten, and its properties cannot be extrapolated from those of more dilute solutions (Singh and MacRitchie 2001). This non-linear change may be reflected in Fig. 6, where the, dough threshold seems to be $\rho >3$ nm$^{-3}$, but the data is too noisy to be decisive. The outlier for $\rho =1$ nm$^{-3}$ is the result of a knotted initial conformation in one of the simulations. The rising number of entanglements is consistent with the observation that the contacts become more non-local for higher densities, as the radial distribution function *g*(*r*) shows lower values for smaller *r* when computed for $\rho =4$ nm$^{-3}$ (as compared to 3 nm$^{-3}$, see Fig. 7).

The density profiles in Fig. 5 were computed only for the last frame of simulations because averaging over many frames made all the profiles look the same (positions of the cavities changed, so after a sufficiently long time, the average density profile smoothed out).

**Fig. 8:**
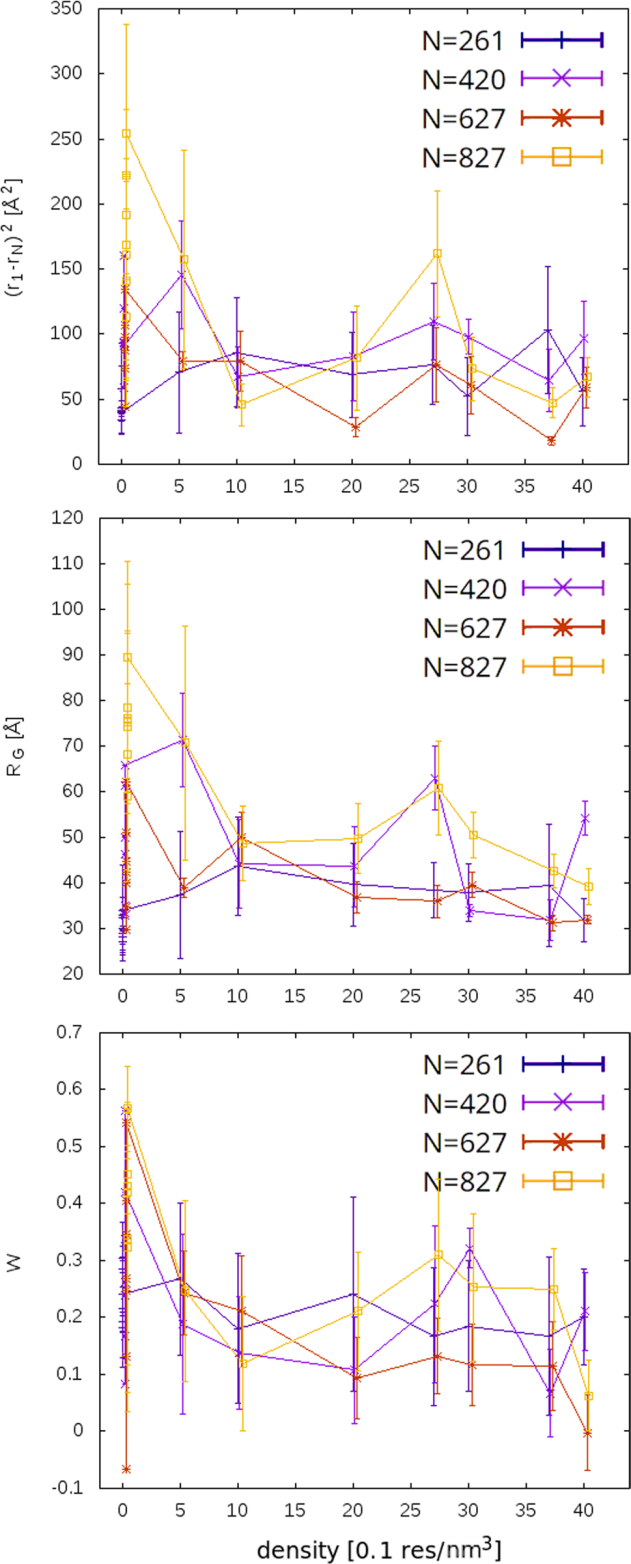
Density dependence of end-to-end distance (top), radius of gyration (middle), and asphericity parameter W (bottom) for 4 chosen chains (the legend shows numbers of residues *N* for each chain). The density zero corresponds to free chains

Figure 8 suggests that concentrated chains are more compacted than when they are in a dilute solution (the density 0 corresponds to the infinitely dilute solution—in practice, simulating a single chain). The variance of the values is also lower for the higher densities (as indicated by the error bars), showing the higher rigidity of the chains in the concentrated protein network as opposed to free chains in the solution.

## Conclusions

For small $\rho$, the volume occupied by the solvent is bigger than the volume of all the cavities (top left corner of Fig. 4). As $\rho$ rises, the solvent starts to be present only in the cavities, which span the entire simulation box (top right and bottom left corners of Fig. 4). Both of these situations correspond to a typical protein solution: $\rho$ should be directly proportional to the concentration *c* for such cases. For even higher $\rho$, the cavities start to get smaller (bottom right corner of Fig. 4). It would correspond to water getting expelled from a real system. It may correspond either to a very concentrated protein solution or to a gel where the solvent is a swelling agent. One can distinguish between these cases by examining the strength of inter-chain interactions. If the proteins remain connected after increasing the simulation box volume, it is a gel (or a protein liquid, if it does not retain shape). A further increase of $\rho$ would lead to either an extremely concentrated solution, a gel with small solvent content or a solid—the last option means structures with no empty (in reality solvent-filled) spaces inside (e.g., amyloid fibers, see Mioduszewski and Cieplak 2020). Of course a protein liquid, gel, or solid may form for smaller $\rho$ (when the phase separation occurs). It should be noted that the volume occupied by the solvent is bigger than the red and pink areas in Fig. 4 because structured water molecules can form a hydration layer close to the residues (Ahlers et al. 2021).

To sum up, 4 different density regimes can be distinguished:

1. The infinitely dilute regime, where each chain is separate.
2. The dilute regime, where chains freely diffuse in the simulation box, they can collide with each other and form dimers, trimers, etc. (a slab-like geometry as used by Dignon et al. 2018 or sufficiently long simulation time may lead to phase separation even in this phase). This is for $\rho < 1$ nm$^{-3}$.
3. The intermediate regime, where chains are interconnected but they do not form a homogeneous system. Large cavities are present, but the network is held together by chain attraction and entanglements. This is for 1 nm$^{-3}< \rho < 3$ nm$^{-3}$. It may be a concentrated solution (that may consist of multiple phases) or a gel, dependent on the system studied.
4. The dense regime, where there are many small cavities, but the system is homogeneous. This is for 3 nm$^{-3} < \rho$. Only one phase (liquid, gel, or solid) is present in this regime.

The threshold values may depend on the type of the system considered, and these values are valid only for the gluten proteins with a certain composition (Mioduszewski and Cieplak 2021b). Gluten proteins are long and attract each other by hydrogen and disulfide bonds, so those findings should be valid only for similar systems. If a phase separation occurs, protein concentration will usually be different between the phases. Short or charged proteins may exhibit a different behavior and require a different treatment. In the Supplementary Information, the percolation theory and chain clustering methods are applied to polyglutamine chains, which are not charged, but are substantially shorter than gluten proteins (Mioduszewski and Cieplak 2020).

If one wants to simulate a protein liquid, gel, or solid, simulations should be performed in the dense regime (unless large computational resources can be spent to wait for a protein network to spontaneously form in a lower $\rho$), but the intermediate regime may be better for simulating systems with high water content or multiple phases (in an explicit solvent simulations, these large cavities would be filled with water). 

### Supplementary Information

Below is the link to the electronic supplementary material.

Supplementary file 1 (pdf 195 KB)

## Data Availability

Data sets generated during the current study are available from the corresponding author on reasonable request. The simulation program (together with usage examples) is available online: https://vitreusx.github.io/pas-cg/ (C++ version) and https://codeocean.com/capsule/9528010 (Fortran version)
