# Supplementary material for: Choosing the right density for a concentrated protein system like gluten in a coarse-grained model
Source: Eur Biophys J. 2023 Jun 28;52(6-7):583–91. doi: 10.1007/s00249-023-01667-8 (PMC10618313; doi:10.1007/s00249-023-01667-8)
Supplement: Supplementary file 1 — (pdf 195 KB) [file 249_2023_1667_MOESM1_ESM.pdf]

Choosing the right density for a concentrated protein system like gluten in a coarse grained model Supplementary Information

Łukasz Mioduszewski[1*]

[1]Faculty of Mathematics and Natural Sciences, Cardinal Stefan Wyszyński University, Wóycickiego 1/3, 01-938 Warsaw, Poland

1 Polyglutamine simulations

The main manuscript describes simulations of gluten. In the Supplementary Information a different system is presented: polyglutamine chains with chain lengths 20, 40 and 60 residues were simulated in order to study polyglutamine aggregation and droplet formation [1]. The simulation box had periodic boundary conditions in all three dimensions and after the initial squeezing the size of the box was constant (no deformation occurred). All the data presented here was gathered in a 500 000 τ period after squeezing and equilibration of the system, which took longer (1 000 000 τ). The number of chains is denoted as N. The total size of the system was 1800 residues ($N = 90$ chains of Q_{20}, $N = 45$ chains of Q_{40} or $N = 30$ chains of Q_{60}).

2 Clusterization

In the DSB model [2] the sidechain-sidechain interaction between glutamine residues is modelled by a Lennard-Jones potential with the minimum $r_0 = 8.63$ Å. If two residues are closer than r_0, they are considered connected. A pair of protein chains is connected if at least one pair of their residues is connected. A cluster is a set of proteins that are connected: all the chains from the set are connected with at least one other chain from the set and with no chains from outside of the set. If such a set cannot be divided into smaller sets with those properties, it is called a cluster. The number of chains in a cluster is called the cluster size.

We can plot probability distributions of cluster sizes. Cluster size 1 means a monomer, cluster size equal to N means that all chains are connected. An example of such distribution is shown on Fig. 1 from the article about polyglutamine simulations [1]. An unimodal distribution with a single

*E-mail: l.mioduszewski@uksw.edu.pl

maximum around 1 means that most of the chains are monomers and the system is in the dilute regime. A bimodal distribution means that some chains are monomeric, and some are in bigger clusters (the intermediate regime). An unimodal distribution with a single maximum around N means that the system is connected and is in the dense regime (but we get no information about its homogeneity). In practice, determining whether the probability distribution is bimodal or unimodal requires making a histogram of cluster sizes (taking cluster sizes from simulation snapshots taken at time interval 5000 τ). Fig. 1 shows the results of classifying such histograms made for the Q_{20} system for different values of temperature T and density ρ. For higher temperatures, the thresholds between the dilute, intermediate and dense regimes are 0.75 nm^{-3} and 1.4 nm^{-3}, respectively. When the temperature is lowered, the system undergoes a phase transition into an amyloid glass phase [1], and the intermediate regime covers a larger range of densities (which means that proteins form several big amyloid-like clusters). A few red dots for high densities indicate systems that formed two or three big clusters with different sizes.

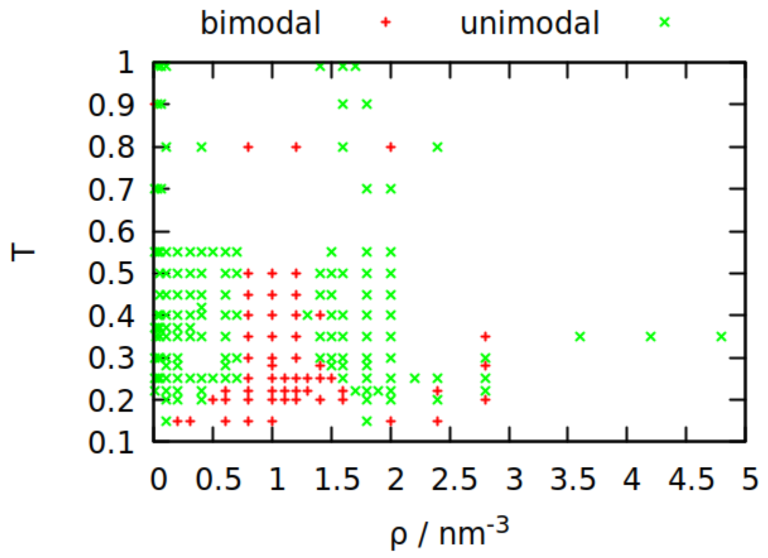

Figure 1: Classification of cluster size histograms into bimodal and unimodal for the Q_{20} system for different values of temperature T and density ρ.

3 Percolation

For polyglutamine simulations percolation can be defined as a state where all protein chains are in one cluster. In one snapshot the system may be in that state, but in another snapshot taken at a different time it may be not. Thus we can calculate the probability P of a system being in the percolation state (P equal to 1 means that it is always the case). Fig. 2 shows plots of $P(T, \rho)$. The threshold between $P \approx 0$ and $P \approx 1$ defines the border between the dilute and dense regime. For Q_{20} it occurs for $\rho \approx 0.75$ nm^{-3}, which corresponds to the dilute-intermediate threshold from

the cluster size analysis (see Fig. 1). For Q_{40} and Q_{60} systems the border is not well defined: few large clusters bounce off each other, randomly and temporarily reaching the percolation state.

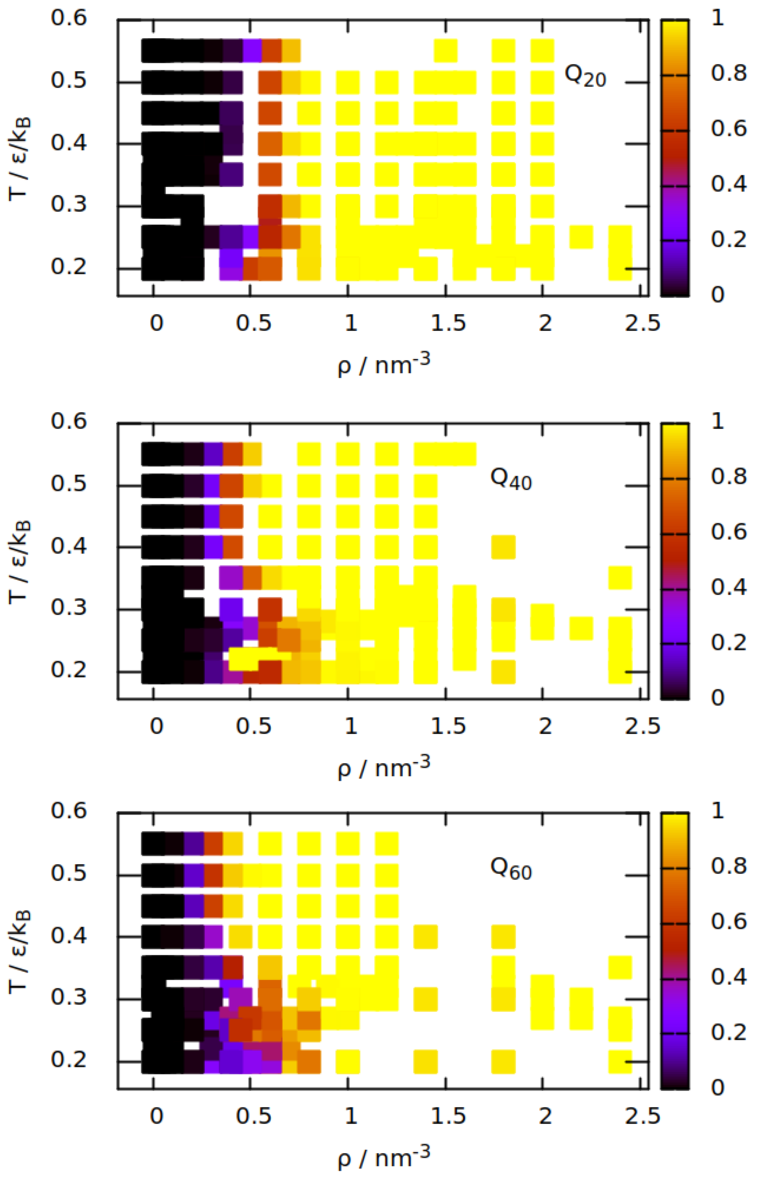

Figure 2: Probability of percolation (whether all chains are connected, see the text) for Q_{20}, Q_{40} and Q_{60} systems, as a function of the temperature T and the system density ρ.

References

[1] Ł. Mioduszewski and M. Cieplak. Protein droplets in systems of disordered homopeptides and the amyloid glass phase. *Phys. Chem. Chem. Phys.*, 22:15592–15599, 2020.

[2] Łukasz Mioduszewski and Marek Cieplak. Disordered peptide chains in an α-c-based coarse-grained model. *Phys. Chem. Chem. Phys.*, 20:19057–19070, 2018.
